# Exosomal Epigenetics

**DOI:** 10.14806/ej.29.0.1049

**Published:** 2024-05-22

**Authors:** Eleni Papakonstantinou, Konstantina Dragoumani, George P Chrousos, Dimitrios Vlachakis

**Affiliations:** 1 Laboratory of Genetics, Department of Biotechnology, School of Applied Biology and Biotechnology, Agricultural University of Athens, Athens, Greece; 2 University Research Institute of Maternal and Child Health & Precision Medicine, and UNESCO Chair on Adolescent Health Care, National and Kapodistrian University of Athens, “Aghia Sophia” Children’s Hospital, Athens, Greece; 3 School of Informatics, Faculty of Natural & Mathematical Sciences, King’s College London, London, U.K.

## Abstract

Epigenetics is the study of heritable changes in gene expression that occur without changes to the underlying DNA sequence. Epigenetic modifications can include DNA methylation, histone modifications, and non-coding RNAs, among others. These modifications can influence the expression of genes by altering the way DNA is packaged and accessed by transcriptional machinery, thereby affecting cellular function and behavior. Epigenetic modifications can be influenced by a variety of factors, including environmental exposures, lifestyle factors, and aging, whilst abnormal epigenetic modifications have been implicated in a range of diseases, including cancer, neurodegenerative disorders, and cardiovascular disease. The study of epigenetics has the potential to provide new insights into the mechanisms of disease and could lead to the development of new diagnostic and therapeutic strategies. Exosomes can transfer epigenetic information to recipient cells, thereby influencing various physiological and pathological processes, and the identification of specific epigenetic modifications that are associated with a particular disease could lead to the development of targeted therapies that restore normal gene expression patterns. In recent years, the emerging role of exosomal epigenetics in human breast milk, highlighting its significance in infant nutrition and immune development. Milk exosomes are shown to carry epigenetic regulators, including miRNAs and long non-coding RNAs, which can modulate gene expression in recipient cells. These epigenetic modifications mediated by milk exosomal RNAs have implications for the development of the gastrointestinal tract, immune system, and metabolic processes in infants.

## Introduction

Exosomal epigenetics refers to the study of how epigenetic modifications, which are chemical changes to DNA and associated proteins that regulate gene expression, can be transferred between cells via exosomes. Exosomes are small extracellular vesicles that are released by many different types of cells and contain a variety of bioactive molecules, including DNA, RNA, and proteins (Foo et al., 2021). Exosomes can play a role in epigenetic regulation by carrying epigenetic information between cells (Qian et al., 2015). For example, exosomes released by cancer cells have been found to contain DNA methylation and histone modifications that can be taken up by recipient cells and alter gene expression patterns (Behbahani et al., 2016). Similarly, exosomes released by stem cells have been shown to contain miRNAs that can regulate gene expression in recipient cells (Foo et al., 2021). Understanding the role of exosomal epigenetics in health and disease has the potential to provide new insights into the mechanisms underlying cellular communication and could lead to the development of new therapeutic approaches.

Exosomal epigenetic modifications refer to the changes in the epigenetic state of cells that are mediated by exosomes. Exosomes can carry various epigenetic modifications such as DNA methylation, histone modifications, and non-coding RNAs that can regulate gene expression in recipient cells (Zhang et al., 2019). For example, exosomes released by cancer cells have been found to contain DNA methylation and histone modifications that can be taken up by recipient cells and alter gene expression patterns, leading to tumor growth and progression (Behbahani et al., 2016).

## Exosomal epigenetic modifications

Exosomal epigenetic modifications refer to the chemical changes to DNA and associated proteins that regulate gene expression, which can be transferred between cells via exosomes (Qian et al., 2015). There are several types of epigenetic modifications that can be transferred via exosomes, including DNA methylation, histone modifications, and non-coding RNAs such as miRNAs, that can influence gene expression, and as a result, they have the potential to contribute to a range of physiological and pathological processes (Zhao et al., 2023). When exosomes are taken up by recipient cells, the miRNAs and mRNAs they contain can influence gene expression patterns in the recipient cell, leading to changes in cellular behavior and function (Valadi et al., 2007). For example, exosomes released by stem cells have been shown to contain miRNAs that can promote cell proliferation and inhibit apoptosis in recipient cells, leading to tissue repair and regeneration (Foo et al., 2021). Similarly, exosomes released by cancer cells can contain miRNAs and mRNAs that promote tumor growth and invasion by regulating the expression of genes involved in cell proliferation, migration, and invasion. In addition to cancer, exosomal epigenetic modifications have been implicated in a range of other diseases, including neurological disorders, cardiovascular disease, and autoimmune diseases; for instance, exosomes derived from mesenchymal stem cells have been shown to promote neuronal differentiation and neurite outgrowth by transferring miRNAs to recipient cells (Zhao et al., 2023).

Exosomal DNA methylation has been shown to play a role in cancer progression, with cancer cells transferring hypermethylated DNA fragments to neighboring cells via exosomes, leading to the silencing of tumor suppressor genes in recipient cells (Behbahani et al., 2016). Additionally, exosomal histone modifications have been implicated in regulating gene expression during embryonic development, and abnormal levels of histone modifications in exosomes have been associated with various diseases, including cancer and neurodegenerative disorder (Volker-Albert et al., 2020). Getting insights in the exosomal epigenetic modifications has the potential to provide new insights into the mechanisms of cellular communication.

### Exosomal gene regulation

Exosomal gene regulation refers to the process by which genetic material carried by exosomes, such as miRNAs, mRNAs, lncRNAs, and other non-coding RNAs, can influence gene expression in recipient cells. Exosomes released by the parent cells can contain various biomolecules, including genetic material (Kalluri and LeBleu, 2020). When exosomes are taken up by recipient cells, the genetic material they contain can regulate the expression of genes in the recipient cell by targeting specific mRNA transcripts and influencing the stability and translation of these transcripts (Lloret-Llinares et al., 2018). For example, exosomal miRNAs have been shown to regulate a variety of cellular processes, including cell proliferation, apoptosis, and differentiation, by targeting specific mRNAs and suppressing their expression (Hu et al., 2012). Similarly, exosomal mRNAs have been shown to be translated in recipient cells, leading to the expression of proteins that can influence cell behavior and function (Hu et al., 2012). The role of exosomal gene regulation in health and disease is an area of active research, with potential implications for the development of new therapeutic strategies. For example, exosomes carrying specific miRNAs could be used to deliver targeted therapies for diseases such as cancer, while exosomes carrying mRNAs could be used to promote tissue repair and regeneration (Fang et al., 2022).

### Exosomal Biomarkers

The use of exosomal biomarkers for disease diagnosis and monitoring has several advantages over traditional biomarkers, such as their stability in body fluids and their ability to be isolated from a variety of sources, including blood, urine, and saliva, and they have been studied as potential indicators of cancer, neurodegenerative disorders, and cardiovascular disease (Huda et al., 2021). In cancer, exosomes have been shown to carry specific proteins, such as CD63 and CD81, that are often upregulated in cancer cells, as well as specific miRNAs and mRNAs that can be used to monitor disease progression and treatment response (Mathew et al., 2021). Exosomal epigenetic information can be transferred between cells and influence gene expression in recipient cells. For example, exosomes have been shown to carry DNA fragments that are hypermethylated at specific sites, leading to the silencing of tumor suppressor genes in recipient cells (Aslan et al., 2019). Similarly, exosomal miRNAs can regulate gene expression in recipient cells by binding to specific mRNA transcripts and regulating their stability and translation (Foo et al., 2021).

In addition to DNA and miRNAs, exosomes can also carry other epigenetic factors, such as histone modifications and other non-coding RNAs. These factors can influence gene expression in recipient cells by altering the accessibility of DNA to transcriptional machinery or by regulating the stability and translation of mRNA transcripts (Zhang et al., 2019). In addition to their diagnostic and prognostic potential, exosomal biomarkers also have the potential to be used as therapeutic targets. For example, exosomes carrying specific miRNAs or proteins could be targeted to inhibit disease progression or promote tissue repair (Aslan et al., 2019; Mathew et al., 2021).

## Epigenetic Effects of Human Breast Milk Exosomes

Human breast milk is an intricate fluid teeming with a multitude of compounds essential for infant nutrition and the development of their immune systems. Among its constituents are secretory immunoglobulins (IgA), leucocytes, lysozyme, and lactoferrin, all of which play crucial roles in conferring passive immunity to infants and regulating the development of their immune systems (Kim and Yi, 2020). Beyond its nutritional value, breast milk contains a rich array of exosomes, which play a crucial role in intercellular communication and the transfer of bioactive molecules between maternal mammary epithelial cells and infant cells (O’Reilly et al., 2021). Emerging evidence suggests that exosomes present in human breast milk carry epigenetic information through the delivery of miRNAs, DNA fragments, and histones to recipient cells, that can influence gene expression and developmental programming in the infant (Leroux et al., 2021).

The transfer of epigenetic information via exosomal cargo is thought to play a critical role in infant health and development. MiRNAs encapsulated within exosomes have been shown to regulate gene expression by targeting specific mRNA transcripts in recipient cells. These miRNAs can influence various cellular processes, including immune function, metabolism, and neuronal development, thereby shaping the developmental trajectory of the infant (Abeysinghe et al., 2020; Zhou et al., 2012). Additionally, exosomal DNA fragments and histones can be transferred to infant cells, where they may contribute to epigenetic modifications and gene regulation. DNA methylation patterns carried by exosomes may influence the establishment of DNA methylation profiles in the infant’s genome, potentially impacting gene expression and long-term health outcomes (Takahashi et al., 2017).

Early-life exposures to maternal exosomal miRNAs and epigenetic regulators could shape the developmental trajectory of key physiological systems, potentially influencing susceptibility to chronic diseases, such as obesity, diabetes, and cardiovascular disorders, in adulthood (Rashidi et al., 2022). The epigenetic effects of milk exosomal RNAs, particularly miRNAs, play a crucial role in promoting intestinal health and immune regulation in infants (Alsaweed et al., 2015). Studies have shown that milk exosomes and their RNA cargoes can enhance intestinal epithelial cell growth and protect against intestinal injury and inflammation (Zeng et al., 2021). For instance, miRNAs such as miR-200a-3p, miR-4334, miR-219, and miR-338 have been found to mitigate intestinal inflammation and damage by targeting proinflammatory genes and pathways (Sun et al., 2013; Xie et al., 2019). Moreover, milk exosomal miRNAs are implicated in immune modulation, with reports suggesting their potential role in regulatory T-cell induction and immune protection (Admyre et al., 2007). Various immune-related miRNAs abundant in milk exosomes have been shown to regulate processes such as B-cell tolerance, plasma cell differentiation, and cytokine expression, thereby influencing immune responses in infants (Chen et al., 2014; Mourtada-Maarabouni et al., 2008; Quan et al., 2020).

Milk exosomal RNAs, including miRNAs and lncRNAs, contribute to epigenetic regulation by targeting genes involved in DNA methylation and histone modification. For example, miRNAs such as miR-148a, miR-152, and miR-29b target DNA methyltransferases, potentially affecting genomic DNA methylation patterns and gene expression (Melnik and Kakulas, 2017). These epigenetic modifications mediated by milk exosomal RNAs have implications for the development of the gastrointestinal tract, immune system, and metabolic processes in infants. While milk exosomal RNAs offer potential benefits for intestinal health and immune function, their implications in metabolic diseases have also raised concerns. Some miRNAs abundant in milk exosomes, such as miR-148a, miR-29b, and miR-21, have been associated with adipogenesis, insulin resistance, and osteoporosis, raising questions about their impact on metabolic health in recipients (Bian et al., 2015; Guglielmi et al., 2017; Monda et al., 2013).

Breast milk contains miRNAs (miRNAs) that are pivotal in orchestrating gene expression in infants, with recent research shedding light on a subset of miRNAs termed xeno-miRNAs (xenomiRs) (Zhang et al., 2012). XenomiRs originate from non-human sources, primarily maternal diet, and are present in human circulation, exerting regulatory effects on gene expression and potentially influencing the immune system. The composition of breast milk miRNAs, including xenomiRs, is intricately linked to maternal dietary intake, highlighting the profound impact of maternal nutrition on infant health outcomes (Stephen et al., 2020). Through vertical transmission via breast milk, xenomiRs derived from dietary sources may modulate gene expression in infants, offering a fascinating glimpse into the intricate interplay between maternal diet and infant health. The presence of xenomiRs in breast milk underscores the importance of considering dietary factors in shaping infant immunity and underscores the complex dynamics of cross-species communication (Liao et al., 2017).

This emerging field of research on milk exosomics not only deepens our understanding of the role of breast milk in infant nutrition and immune development but also opens new avenues for optimizing infant health through targeted nutritional interventions.

## Conclusion

Recent research has focused on exploring the role and applications of exosomes, particularly in human breast milk, to elucidate their epigenetic effects and mechanisms underlying exosomal epigenetic regulation in health and disease. Promoting breastfeeding initiation and duration can yield far-reaching benefits for infant health and development due to the diverse array of bioactive components, including exosomes, present in human breast milk. Additionally, efforts to optimise maternal nutrition and lifestyle factors during lactation may enhance the composition and functionality of exosomes in breast milk, further augmenting their epigenetic effects on infant health.

While substantial progress has been made in elucidating the epigenetic effects of exosomes in human breast milk, several questions remain unanswered. Future research endeavors should focus on characterizing the epigenetic effects of exosomes in breast milk, delineating their mechanisms of action, and discerning their long-term effects on infant health and disease susceptibility. By unraveling the mechanisms underlying the transfer and impact of exosomal cargo in breast milk, the scientific community aims to harness this knowledge to devise innovative strategies for promoting infant health and disease prevention.

## Key Points

- Elucidating the mechanisms by which exosomes facilitate the transfer of epigenetic cues between cells, modulating gene expression patterns and cellular responses.
- The influence of exosomal epigenetic cargo present in human breast milk on neonatal health and developmental outcomes, with a focus on immune modulation and metabolic regulation, is investigated.
- Exosomal biomarkers have a diagnostic, prognostic, and therapeutic potential for various diseases, including their utility in targeted therapeutic interventions.
- Evaluating the impact of maternal dietary microRNA content in breast milk on neonatal gene expression and immune function is key modulator in shaping neonatal health.
